# On the influence of density and morphology on the Urban Heat Island intensity

**DOI:** 10.1038/s41467-020-16461-9

**Published:** 2020-05-27

**Authors:** Yunfei Li, Sebastian Schubert, Jürgen P. Kropp, Diego Rybski

**Affiliations:** 1 Potsdam Institute for Climate Impact Research – PIK, Member of Leibniz Association, P.O. Box 60 12 03, Potsdam, 14412 Germany; 2 Institute for Environmental Science and Geography, University of Potsdam, Am Neuen Palais 10, 14469 Potsdam, Germany; 3 Geography Department, Humboldt-Universität zu Berlin, Unter den Linden 6, 10099 Berlin, Germany; 4 Department of Environmental Science Policy and Management, University of California Berkeley, 130 Mulford Hall #3114, Berkeley, CA 94720 USA

**Keywords:** Climate sciences, Statistical physics

## Abstract

The canopy layer urban heat island (UHI) effect, as manifested by elevated near-surface air temperatures in urban areas, exposes urban dwellers to additional heat stress in many cities, specially during heat waves. We simulate the urban climate of various generated cities under the same weather conditions. For mono-centric cities, we propose a linear combination of logarithmic city area and logarithmic gross building volume, which also captures the influence of building density. By studying various city shapes, we generalise and propose a reduced form to estimate UHI intensities based only on the structure of urban sites, as well as their relative distances. We conclude that in addition to the size, the UHI intensity of a city is directly related to the density and an amplifying effect that urban sites have on each other. Our approach can serve as a UHI rule of thumb for the comparison of urban development scenarios.

## Abstract (web summary)

How UHI intensity responds to variations of urban structure is unclear. Here the authors proposed a reduced form approach that is able to estimate UHI intensities based only on the number and location of urban sites as well as their distance.

## Introduction

The canopy layer urban heat island (UHI) effect, a phenomenon manifested by elevated near-surface air temperatures in cities compared to their non-urban surroundings, is mainly due to land surface modification in connection with urbanisation. The surface energy balance of urban areas differs from that of vegetated land in various aspects. First, as impervious surfaces replace natural land cover of low albedo, high thermal capacity, and high thermal conductivity, urban areas exhibit reduced latent heat flux and increased heat absorption^1^. Second, the geometry of urban surfaces featuring buildings and street canyons leads to reduced overall wind ventilation as a consequence of increased roughness, and to more radiation trapping due to in-canyon reflections^2,3^. Anthropogenic heat release from human activities also adds to the accumulation of heat. The combined effect of these properties causes the UHI phenomenon. Usually, the UHI intensity peaks at night^4^ as heat stored in the urban surfaces during daytime is released^5^, resulting in a lower cooling rate compared to vegetated surfaces.

The UHI effect has various direct and indirect impacts on urban dwellers and their health^6–8^. In many cities, it exposes urban dwellers to extra heat stress and thus leads to thermal discomfort, as well as heat-related health problems during hot summer days^9,10^. In particular during heat wave events, the risk of heat morbidity and mortality increases^9,11–13^ as the UHI effect interacts with heat waves by prolonging and intensifying hot conditions^11,14,15^. Warming enhanced by urbanisation has been identified in many cities and regions^10,16–18^, which implies more severe heat stress in the future.

The impacts are further exacerbated when taking climate change into consideration^2,11,19^, though they interact non-linearly and are found to produce warming that is less than the simple sum of their individual contributions^20^. Apart from health risks, the joint economic costs of urban impacts from the UHI effect and climate change have been estimated to be 2.6 times those without UHI effect^21^. Although UHIs do not remain stable under climate change^2,22^ or urban development^20^, future strong nocturnal warming due to urban effects has been found in many cities^10,11,14,18,20^. This may not be too critical under normal temperature conditions. However, during heat waves this aggravated heat stress can create significant risks to urban residents, as mortality risk is found to be significantly associated with minimum temperatures^23,24^. Therefore, measures to reduce the impact of UHI will also contribute to urban heat stress mitigation, especially in the future with more frequent and stronger extreme heat events due to the interactions between urban climate, heat waves, climate change, and urbanisation.

Many studies on the neighbourhood or block scale have related higher temperature to urban characteristics, such as impervious surface fraction (or its opposite, nature surface fraction), building density^13^, and street canyon aspect ratio^4,25^. Some researchers have also tried to quantify the neighbourhood-scale UHI intensity based on this knowledge^26,27^. Those findings not only help to advance our understanding of the physical mechanism behind the UHI formation, but also shed light on useful measures to alleviate heat stress in hotspots, or to generally create a better thermal environment. Benefits of some local interventions like increasing vegetation space^28^, green roofs^29^, and cool coating of buildings and infrastructure^30^ have been proven by both numerical studies and practical applications.

However, such mitigation strategies have very local influences on climate^31^ and may not always work as efficiently at night as during hot afternoon^20^. Besides, many aspects of urban form (such as overall dimensions, skyline and poly-centricity, sprawling, and compactness)^1^ can affect the spatial pattern of urban climates^31,32^. This suggests the potential of urban form and structure to mitigate urban heat stress. For example, urban characteristics measured by a sprawling index were found to be strongly related to the growth rate of extreme heat event frequency^33^. Thus, rapid urban growth poses challenges to urban heat mitigation, but it also presents an important opportunity to implement urban climate knowledge in newly developing areas. A quantitative assessment is needed to support urban decision making that takes the UHI effect into account. In this work, we quantify the relationship between UHI and the urban form, namely the three-dimensional configuration of urban elements. With this, urban growth can be developed in a way that does not create a strong UHI effect in the first place instead of reducing the urban heat after expanding. As the UHI effect has the greatest impact during heat waves and usually reaches its maximum at night time, we focus on these conditions.

Here, we simulate the urban climate of hypothetical cities with variable size, density, and compactness/sprawling. To this end, we use the surface and vegetation characteristics of the region around Berlin (Germany), replace the city with generated clusters, run the urban climate model driven by the same lateral climate conditions, and extract the UHI intensity. By repeating the procedure for different mono-centric clusters, we infer an expression for the UHI intensity that is solely based on the area and the gross building volume. By studying a wider range of city shapes, we generalise and propose a reduced form to estimate UHI intensities based only on the structure of urban sites, as well as their relative distance. We conclude that in addition to the size, the UHI intensity of a city is directly related to the building density, and an amplifying effect that urban sites have on each other.

## Results

### Modelling set-up and UHI intensity definition

**Fig. 1:**
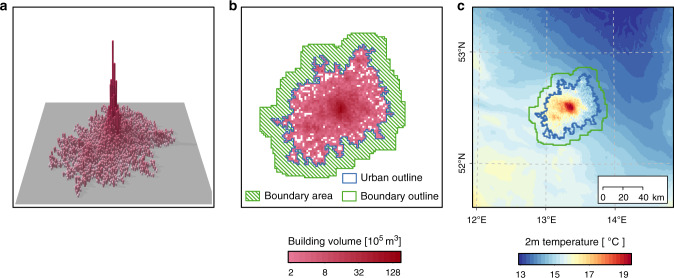
Example of generated urban clusters and resulting heat patterns. **a** Three-dimensional illustration of average building height in each grid cell of a considered cluster. **b** Gross building volume in the cluster together with cluster edge and surrounding boundary. **c** Simulated temperature field at night (02:00 local time). The UHI intensity is defined as Δ*T* = 〈*T*_C_〉 − 〈*T*_B_〉, where 〈*T*_C_〉 and 〈*T*_B_〉 are the average 2-m air temperatures in the city (blue line) and boundary (green line), respectively.

We start by generating urban clusters resembling real-world cities and define the urban canopy parameters (UCP) accordingly. Then, we use the physical characteristics of the region around Berlin to simulate the urban climate employing the COSMO-CLM/Double Canyon Effect Parametrization Scheme (CCLM/DCEP) urban climate model^34^, always driven by the same lateral climate conditions (see “Methods” section). An example, urban cluster and its building information is illustrated in Fig. 1a. With each configuration, we simulate the 2-m air temperature in the period of August 1st–7th, 2003, i.e., during a heat wave characterised by predominately clear skies and light winds^28^. In order to analyse the overall UHI intensity of the city, we consider the urban cluster and a non-urban belt with approximately equal area (Fig. 1b), as used for remote sensing data^35,36^. Then, we define the hourly UHI intensity Δ*T*_*i*_ as the difference between the average 2-m air temperatures in both areas, i.e., $\Delta {T}_{i}=\langle {{T}_{{\rm{C}}}}_{i}\rangle -\langle {{T}_{{\rm{B}}}}_{i}\rangle$, where $\langle {{T}_{{\rm{C}}}}_{i}\rangle$ and $\langle {{T}_{{\rm{B}}}}_{i}\rangle$ are the average temperatures at local time *i* in the cluster and the boundary, respectively (see Fig. 1c). We extract the daily maximum UHI magnitude, and average it over 7 days for each simulation. For simplicity, UHI intensity and Δ*T* henceforth refer to the 7-day-average maximum UHI magnitude based on 2-m air temperature difference, unless otherwise indicated. A more general discussion of the UHI intensity and shortcomings of how to measure it can be found in previous studies^37,38^. Finally, we build models expressing Δ*T* as a function of building parameters.

### Simulations with mono-centric urban clusters

**Fig. 2:**
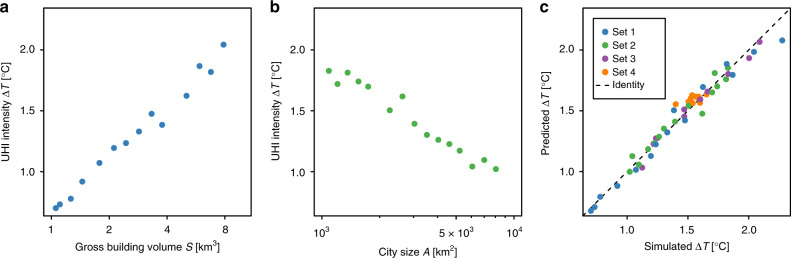
UHI intensity as a function of city characteristics and fitting performance of Eq. (1). **a** The UHI intensity is plotted vs. gross building volume for constant city size. **b** The UHI intensity is plotted vs. city size for constant gross building volume. **c** Predicted and simulated UHI intensities are plotted against each other. Supplementary Fig. 1 illustrates the various sets of generated clusters.

We repeat the analysis for 50 clusters (generated by a gravitational urban growth model^39,40^, see “Methods” section) that vary by size and compactness. In the Supplementary Fig. 1, we provide details on the clusters. The clusters are characterised by their size *A* (km^2^), which is given by the number of urban cells, and by the gross building volume *S* (km^3^), which is given by the sum of building volumes *w*_*i*_ over all urban cells *i* (for calculation of *w*_*i*_, see “Methods” section). Assuming constant floor height and constant floor area per person, *S* is proportional to the population size of the entire city. If we keep the cluster size constant, we can study how the UHI intensity depends on the gross building volume. As shown in Fig. 2a, Δ*T* increases approximately linearly with ${\mathrm{ln}}\,S$. Analogously, we can keep the gross building volume constant and test how the UHI intensity depends on the cluster size (Fig. 2b). In this case, Δ*T* decreases approximately linearly with ${\mathrm{ln}}\,A$. It is plausible that given the same urban area, cities with higher density exhibit more pronounced UHI intensities and less dense cities exhibit reduced UHI intensities^2,29^.

Combining both results, we find that the UHI intensity can be described by

1 $$\Delta T={a}_{1}{\mathrm{ln}}\,A+{a}_{2}{\mathrm{ln}}\,S+{a}_{3},$$

where *a*_1_, *a*_2_, *a*_3_ are parameters. When fitting this form to all 50 investigated clusters, we obtain *a*_1_ = −0.43 K, *a*_2_ = 0.65 K, *a*_3_ = 3.90 K, and *R*^2^ = 0.96. In Fig. 2c, we plot predicted and simulated UHI intensities against each other. From these results, we conclude that the UHI intensity can be described by a linear combination of logarithmic city area and logarithmic gross building volume (resembling city population), which is a generalisation of previous findings^5,31,36,41–43^. If we introduce the urban density^1,2^ and define it as *S*/*A*, then we can rewrite Eq. (1), $\Delta T=({a}_{1}+{a}_{2})\,{\mathrm{ln}}\,A+{a}_{2}\,{\mathrm{ln}}\,(S/A)+{a}_{3}$ or $\Delta T=-{a}_{1}\,{{\mathrm{ln}}}\, (S/A) + ({a}_{1}+{a}_{2})\,{\mathrm{ln}}\,S+{a}_{3}$, showing that the UHI intensity increases linearly with the logarithm of the density (given *a*_1_ < 0 and *a*_2_ > 0). Moreover, in the Supplementary Fig. 2, we show that according to the simulations the parameters *a*_*i*_ approach 0, with growing street canyon width. Certainly, the parameters *a*_*i*_ also depend on additional factors, in particular the background climate^5,41,44^ (such as wind speed, precipitation, and cloud cover) and thermal properties of the rural surface^2,44,45^.

### Simulations with a wide range of urban forms

One can easily think of configurations where *A* and *S* are unchanged, but the urban form is very different. The investigated urban clusters do exhibit a range of compact or scattered shapes (Supplementary Fig. 1), but more complex spatial features, e.g., as captured by the fractal dimension^31,46^, can hardly be analysed based on those clusters since their fractal dimension covers a comparably small range^39^. Accordingly, we perform further simulations with more extreme urban forms that are beyond real life cities. We generate ten different spatial patterns with a range of sizes and repeat the urban climate simulations. In order to avoid additional complexity, here we use constant building height and canyon width throughout the urban sites and simulations. The shapes considered range from the rather sparse Cantor Dust to the more compact Sierpinski Carpet. In addition to these regular fractals, the patterns also include irregular ones, e.g., diffusion-limited aggregation^46–48^ (DLA) clusters, and non-fractal shapes, such as the filled circle (Supplementary Fig. 3, Table 1).

**Fig. 3:**
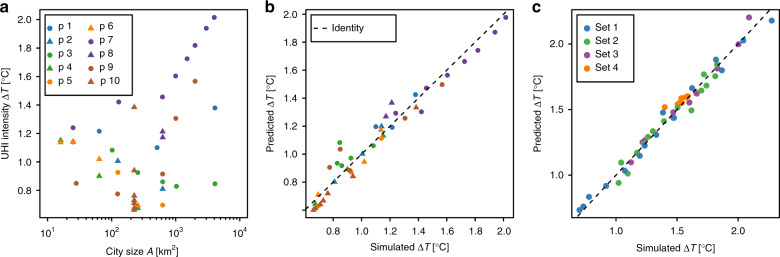
UHI intensity as a function of city size and form attributes. **a** The UHI intensity is plotted against city size of corresponding spatial patterns (Supplementary Fig. 3, Table 1). **b** The UHI intensity predicted by Eq. (2) is plotted against simulated UHI intensity. **c** For the realistic urban clusters (Supplementary Fig. 1), the UHI intensity predicted by Eq. (3) is plotted against simulated UHI intensity.

As the density here held constant, i.e., *A* ~ *S*, it is sufficient to plot Δ*T* as a function of the area *A* if we want to apply Eq. (1). The results are shown in Fig. 3, demonstrating that overall, the UHI intensity tends to increase with the logarithmic size, but that for a given size, the resulting Δ*T*-values spread over a wide range that is certainly due to the variety of shapes that have been used. Although fractal geometry represents a convenient formalism to characterise spatial structures, we found that the fractal dimension is not a sufficient indicator to describe the UHI intensities, and we propose an alternative ansatz as follows. Motivated by the perception that any urban site has a heating influence on other urban cells that declines with the distance between the urban cells, we explore the following educated guess combining a size term and a form term

2 $$\Delta T={b}_{1}{\mathrm{ln}}\,A+{b}_{2}\frac{1}{N}\mathop{\sum }\limits_{j}^{N}\mathop{\sum }\limits_{i\ne j}^{N}{d}_{ij}^{-\delta }+{b}_{3}\quad {\rm{with}}\quad \delta \simeq 3/2,$$

where *d*_*ij*_ is the Euclidean distance (km) between the urban sites *i* and *j*, *N* is the total number of urban cells, and *b*_1_, *b*_2_, *b*_3_ are parameters. If *h*_*ij*_ is the heat influence that site *i* has on *j*, then ${H}_{j} = {\sum }_{i}{h}_{ij}$ is the influence that site *j* receives from all other sites. Since for Δ*T*, we calculate the average over all the cells of a city, we need an additional sum over all sites and a division by their number, i.e., $\frac{1}{N}{\sum }_{j}{H}_{j}$, which with ${h}_{ij} \sim {d}_{ij}^{-\delta }$ corresponds to the second term in Eq. (2). When fitting Eq. (2) to all ten patterns (42 simulations), we obtain *b*_1_ = −0.19 K, *b*_2_ = 0.04 K km^3/2^, *b*_3_ = 1.60 K, and *R*^2^ = 0.95. The performance is visualised in Fig. 3b, where predicted and simulated UHI intensities are plotted against each other. In the Supplementary Fig. 4, we show how we found the value of the exponent *δ* based on the simulations of pattern 10 (Supplementary Fig. 3), i.e., a square of constant *N* but with varying space between the urban pixels. Equation (2) suggests that the distribution of distances between the urban sites contains the information necessary to capture the UHI intensity of basically any urban shape.

### General regression model

A naturally emerging question is if Eq. (2) can also be used to estimate the UHI intensity of the generated urban clusters that led to Eq. (1). In order to unify both approaches, we introduce a weighting factor $f({{f}_{{\rm{u}}}}_{i},{w}_{i},{Y}_{i})$ into the second term of Eq. (2), i.e., as a function of the building volume *w*_*i*_, the urban fraction ${{f}_{{\rm{u}}}}_{i}$, and the street canyon width *Y*_*i*_ for each urban cell. We find reasonable fitting for $f({{f}_{{\rm{u}}}}_{i},{w}_{i},{Y}_{i}) \sim {\big(\frac{{{f}_{{\rm{u}}}}_{i}{w}_{i}}{{Y}_{i}}\big)}^{1/2}$. Moreover, we have to include the ${\mathrm{ln}}\,S$ term as in Eq. (1). Thus, in general,

3 $$\Delta T={c}_{1}\,{\mathrm{ln}}\,A+{c}_{2}\,{\mathrm{ln}}\,S+{c}_{3}D+{c}_{4}\quad {\rm{with}}\quad D=\frac{1}{N}\mathop{\sum }\limits_{j}^{N}\mathop{\sum }\limits_{i\ne j}^{N}{\left(\frac{{{f}_{{\rm{u}}}}_{i}{w}_{i}}{{Y}_{i}}\right)}^{1/2}{d}_{ij}^{-3/2},$$

where *c*_1_, *c*_2_, *c*_3_, *c*_4_ are parameters. Fitting leads to *c*_1_ = −0.26 K, *c*_2_ = 0.28 K, *c*_3_ = 0.07 K km^1/2^, *c*_4_ = 2.43 K, and *R*^2^ = 0.99. In Fig. 3c, we again plot predicted and simulated values against each other, and find similar agreement as in Fig. 2c, despite the more general approach. We would like to note that we also obtain decent fitting if we employ $f({{f}_{{\rm{u}}}}_{i},{w}_{i},{Y}_{i}) \sim {\mathrm{ln}}\,\left(\frac{{{f}_{{\rm{u}}}}_{i}{w}_{i}}{{Y}_{i}}+1\right)$, as the forms of power laws with small exponents and of the logarithmic function are quite similar. We find that regressing $\Delta T={c}_{1}\,{\mathrm{ln}}\,A+{c}_{2}D+{c}_{3}$, i.e., without the ${\mathrm{ln}}\,S$ term, also provides reasonable fitting but Eq. (3) is preferable according to the Akaike Information Criterion^49^. Moreover, Eq. (3) can be rewritten into another form that includes urban street canyon aspect ratio^25,50^ (see Supplementary Note 1). Overall, Eq. (3) represents a comparatively simple way to estimate the UHI intensities based on urban form and size. Given that the parameters *c*_*i*_ are known, all that is necessary to estimate the UHI intensity is the spatial information of urban sites and building heights.

### Application to a real-world example

Here, we briefly illustrate how the above findings can be applied to idealised urbanisation scenarios of the (real) city of Berlin. We restrict the simulations to urban development that takes place vertically (decrease/increase in building height), horizontally (shrinking/expanding extent of urban cluster), or that decreases/increases the urban fraction. For each of these three urbanisation types, we have created several configurations with changes in gross building volume varying between −50% and +100% compared to the present urban canopy data of Berlin (see “Methods” section). The parametrization is then used both to run the urban climate model and to calculate the quantity *D* in Eq. (3). Of course, in addition to urban structure factors, weather conditions (e.g., cloud cover and type, wind speed, and direction), and rural surface conditions (such as thermal admittance and surface wetness) are very important factors influencing the UHI intensity^45^. Together with the reference run (the simulation with real urban canopy data for Berlin that was used to validate the configuration of the climate model, see “Methods” section), we performed 21 simulations.

**Fig. 4:**
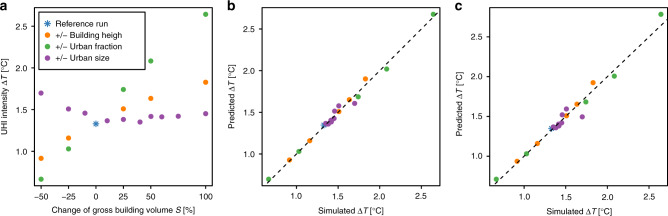
UHI intensity from urbanisation scenarios of Berlin. **a** The UHI intensity is plotted against the gross building volume *S* for corresponding urbanisation scenarios. **b** The UHI intensity predicted by Eq. (3) is plotted against simulated UHI intensity. **c** as **b** but with out-of-sample validation, i.e., the value to be validated is removed from sample and the regression is based on the remaining ones (repeated for each value).

The simulated UHI intensities from these scenarios are plotted against the change in gross building volume in Fig. 4a. We find that the increases in building height and urban fraction both lead to increases in UHI intensity, yet they behave differently, as the latter leads to faster UHI intensity increase. This is probably due to the stronger shadow effects from taller buildings, which reduce heat storage during the day. For the scenarios with changed urban cluster size, the trends are less clear. The fluctuations may be caused by the fact that the randomly removed or added urban cells vary in urban surface fraction, building structure, and street canyon configurations. In addition, the background climate and rural surface characteristics are heterogeneous throughout the domain, so that different expansion directions of the urban cluster will have slightly different influences on the resulting UHI intensity. The higher than reference run UHI intensity of the scenarios with decreased urban cluster size can be understood, when considering that outer cells normally exhibit lower urban surface fraction and building height. When these cells are removed, the remaining central urban core has relatively high building density, leading to higher UHI intensity.

When regressing Eq. (3) on the urban climate results from these scenarios, we obtain *c*_1_ = −0.25 K, *c*_2_ = 0.13 K, *c*_3_ = 0.18 K km^1/2^, *c*_4_ = 1.85 K, and *R*^2^ = 0.99. As for these simulations, we use heterogeneous real-world external data (such as vegetation, orography, and soil parameters, etc.) instead of homogeneous external data for simulations with model generated mono-centric urban structures, the coefficients here differ slightly from before. The predicted and simulated values are plotted against each other in Fig. 4b. Considering the high heterogeneity of the urban structure factors within the real UCP data and the derived scenario UCP data, we can conclude that our general regression form as in Eq. (3) holds not only for model-created urban structures, but also for real-world urban structures. In order to test the robustness of these results, we last perform an out-of-sample validation: we remove one simulation from the 21 samples, regress Eq. (3), and obtain a prediction that is independent from its corresponding simulated value. Then, we repeat the procedure for each sample in the set (leave-one-out cross-validation). Comparing the predicted and simulated values, we can see that the UHI intensities predicted by our approach show agreement with the simulated UHI intensities. Except for two values with a deviation of −0.21 K and 0.14 K, the rest of the predictions have an error within ±0.1 K.

## Discussion

The UHI effect can increase the frequency of extreme heat events, extend the duration of high temperatures, and narrow the time window for relief from high-heat exposure^9,11,14^. This heat stress may deteriorate further when taking climate change and rapid ongoing urbanisation into account. Moreover, as the majority of world’s population already lives in cities, urban areas are expected to absorb the lion’s share of global population growth that is estimated to be 2.2 billion by the end of this century^51^. This means that in the future many more people will be exposed to more frequent and intensified extreme heat events, not to mention demographic change and an increasing proportion of vulnerable elderly people. Therefore, urban development policies need to take the UHI effect into account^21^ and make proper use of effective ways to reduce excessive urban heat.

Achieving this goal requires more comprehensive understanding of how the UHI effect is influenced by key local- and regional-scale factors, such as urban canyon structure, building density, urban surface fraction, and urban form. A challenge in the study of cities is that many factors and characteristics vary among cities, most notably the background climate^5^, thermal properties of the rural surface^45^, city size^41^, density^44^, urban form^52^, street geometry^8,44^, and building material^2,44^. Here, by employing simulated cities, we can keep the climate conditions constant and clearly define these factors, and thus investigate the UHI phenomenon for cities over almost two orders of magnitude.

We quantify the relationship between key urban factors and the UHI intensity, and propose a regression model to quantitatively estimate the UHI intensity based on detailed 3D urban structure data. Our results show that: firstly, given the same urban area or gross building volume, the UHI intensity is strongly influenced by the building density (gross building volume per unit area, calculated as the product of total building plan area, and average building height within an urban area unit); and secondly, increasing building density will lead to stronger UHI intensity. However, due to different effects of aspect ratio, increasing building plan area causes a more rapid increase in UHI intensity than adding vertical building height. Given knowledge about the coefficients, Eq. (3) can serve to quantify the effect of interventions on the city in question and to investigate scenarios of the urban development.

Our results confirm that increasing urban fraction and building height will enhance the UHI intensity, which is consistent with previous studies^4,26–29,41,53^. Although increasing building height means more shading effect and less heat storage within a street canyon during the day, it also leads to larger aspect ratios and lower night time cooling rates due to stronger trapping of outgoing radiation. The extent to which one of these two effects offsets another still requires further investigation. According to our results, increasing the building density through taller buildings leads to slower increase in UHI intensity, and the increase rate gets smaller for larger street canyon aspect ratio^4^. On the other hand, based on numerical modelling, Marciotto et al. found a peak point of aspect ratio at ~3.5, beyond which the maximum UHI intensity will decrease with increasing aspect ratio^25^. This is not a contradiction as in our work, most of the street canyons still have an aspect ratio <3.5 even when the height of all buildings is doubled. However, it is also unrealistic to have a that large average aspect ratio within many grid cells at 1 km resolution.

Regarding the influence of urban form, our results show that the sprawling development will lead to a better thermal environment when considering the entire urban area. Stone et al. found that sprawled cities show a greater rate of increase in the frequency of annual extreme heat events^33^. A reason for this difference could be their use of observational data acquired at weather stations that are often located near the airport instead of the city centre. Moreover, when the urban area and the gross building volume are controlled, the factors within the term *D* in Eq. (3) still interact with each other non-linearly. Under the premise that the street canyon geometry is homogeneous over all urban sites, the term *D* clearly indicates that more compact urban clusters will lead to higher UHI intensities. However, without this precondition the situation is more complex. Future work that links the quantity *D* to factors like urban fractality^31,46,54^, urban centrality/poly-centrism^33,55^, anisometry^31^, and intra-urban street canyon geometry will further our understanding of the influence of the urban form on the UHI effect.

Our results, to some extent, cross the scale hierarchy with regards to urban heat stress mitigation by aggregating the complex interactions of vegetation fraction and canyon geometry at the neighbourhood-scale grid cell (in our case, at the scale of 1 km) into an impact at the city scale. This means that city-scale UHI intensity cannot simply be scaled up from that of the neighbourhood scale, as nearby neighbourhoods also influence each other. Since there is no single best design that meets all climate objectives^1^, a quantitative assessment of the impact of different designs can help to balance between different objectives^30,37,56–58^. However, it is beyond the scope of this paper to integrate our findings into a more holistic frame, since decision making on urban design is a very complex process that requires consideration of many other aspects. For example, a denser city can be preferable regarding energy efficiency but will lead to greater UHI intensity^57^, a proper comparison requires quantitative assessment of both objectives. Even for the same objective of urban heat stress mitigation, it is difficult to clearly prefer one development design over another one before their detailed 3D urban structures are available, or at least some of the factors are fixed. The main reason is that the factors in Eq. (3), in particular building height and street width, interact and impose limits on each other. Instead, our approach permits an assessment^30,56,58^ that takes the 3D urban structures of different urban development scenarios as inputs, and enables the comparison between these scenarios with regard to heat stress mitigation on the city scale.

Some limitations exist in this work and the application of our results. Firstly, besides urban form and factors related to street canyon geometry^1^, weather conditions and rural surface characteristics^44,45^ play an important role in determining UHI intensities. To apply Eq. (3) to another city, one would need to derive the coefficient of the regression again, requiring ~20 simulations. This hampers the fast application of our results, especially for those without expertise in running numerical climate simulations. Properly identifying a representative heat wave event and always using it as standard driving data could help to avoid unnecessary simulations. Secondly, with the coefficients known, applying Eq. (3) still requires detailed 3D urban structure data for the development scenarios under consideration. This data will become increasingly available with the rapid development of spatial information technology. Lastly, for simplification and to better separate the influences of the various factors, we excluded anthropogenic heat in this study. For Berlin, the influence of anthropogenic heat release on nocturnal UHI effect should be relatively small during summertime according to studies on other temperate cities^45,59–61^. Moreover, during the night, anthropogenic heat release from cooling should be negligible as the majority of households do not use air conditioning. However, for cities or scenarios where cooling equipment is widely operated during hot summertime, UHI intensity can be increased by >1 ^∘^C (refs. ^62–64^). Further work on the influence of anthropogenic heat will be helpful for more accurate UHI prediction for cities, where cooling devices are widely used.

## Methods

### Climate model

The mesoscale non-hydrostatic climate model CCLM (ref. ^65^) coupled with a multi-layer urban canopy model (UCM), the DCEP (ref. ^34^), was used in this study. Previous work has shown that diurnal variation and magnitude of UHI can be well represented in CCLM/DCEP during summer months^28,66^.

CCLM was developed from the operational weather forecast Local Model of the German Meteorological Service by the CLM-Community and has been the community model of the German climate research since 2005. In the standard CCLM, cities are represented by a bulk-transfer scheme with modified soil and vegetation parameters. An urban scheme is necessary to represent important urban characteristics in terms of thermal properties and vertical effects of buildings^28^. The DCEP scheme, based on the Building Effect Parametrization^67^, accounts for the effects of buildings and streets configuration on the atmosphere. When coupled with CCLM, DCEP is only applied to the urban fraction of a mesoscale model grid cell, the remaining natural surface fraction is treated by the land surface scheme of CCLM. In DCEP, the urban surface is conceptualised as multiple series of identical street canyon elements that are characterised by canyon direction, street width, building height, and building width. Therefore, UCP required by DCEP for each urban grid cell are: urban surface fraction, canyon direction distribution, building height distribution, street width, and building width.

### Model set-up and data analysis

We conducted a chain of three nested CCLM simulations with resolutions of 0.165^∘^, 0.025^∘^, and 0.009^∘^, see coverage of each model domain in Supplementary Fig. 6b. The domain of the innermost simulation was centred at Berlin. A period of 1 week during a heat wave event was simulated^28^. The coarsest simulation was driven by ERA-Interim reanalysis data with a spin-up time of 5 years. The remaining two nesting steps started 6 months and 12 days, respectively, before the analysed period.

The DCEP scheme is only applied in the finest simulations. To validate our model configuration, we conducted a reference run with UCP data derived from a 3D dataset of Berlin at 1 km resolution^28^. Statistics of the model performance against observational data (measured at six weather stations located in and near Berlin, see Supplementary Fig. 6b) are shown in Supplementary Table 2. In terms of mean error, mean absolute error, and root-mean-square error, the model satisfactorily reproduces the 2-m air temperature.

Configurations for the simulations with generated UCP data were the same with the reference run except that the external data, such as vegetation, orography, and soil parameters were made homogeneous based on the mean value of the finest domain. This minimises the effect of non-urban parameters on our results.

For each generated urban cluster, we define a non-urban boundary of approximately the same area by determining several layers of cells surrounding the urban area^36^. The difference between average 2-m air temperatures of urban area and rural boundary area was taken as the canopy layer UHI intensity.

### Urban growth model

We used a gravitational urban growth model^39^ to create realistic 3D urban canopy data. This model, based on the concept that growth is more likely to take place close to high densities, is capable of reproducing various attributes of real-world cities, such as the radial gradients of population density, radial gradients of urban fraction, and the power law between the population and city area. On a square grid, the probability of growth in cell *i* is given by ${p}_{i}=\frac{G}{{M}_{i}}{\sum }_{j\ne i}{v}_{j}{d}_{i,j}^{-\gamma }$, where *γ* is the main parameter, *d*_*i*,*j*_ is the Euclidean distance from cell *i* to *j*, *v*_*j*_ is the value in cell *j*, *M*_*i*_ is a site-specific normalisation constant^39,40^, and *G* is another parameter determining the overall rate of growth. Starting with a single *v* = 1 cell in the centre, the model is run iteratively incrementing the counts *v*_*i*_ → *v*_*i*_ + 1, if *z* < _*pi*_, where *z* is a random number between 0 and 1. The exponent *γ* controls the shape of the emerging urban clusters, i.e., small *γ* lead to sprawled and radial symmetric structures, and large *γ* lead to compact forms with less radial symmetry. Consistent with ref. ^39^, we explored *γ* ∈ {2.0, 2.05, …, 2.7, 2.75} and 1000 × 1000 system size.

From the resulting 14,1758 clusters, for each *γ* value we first select one cluster with an area of approximate 2000 cells (as we put these clusters in the climate model domain with 1 km^2^ resolution, its area corresponds to 2000 km^2^). The corresponding gross building volume increases with *γ*. We name these clusters set 1, and took the cluster for *γ* = 2.5 in set 1 as reference cluster *C*_ref_. Similarly, we select one cluster for each *γ* value that has approximately the same gross building value as *C*_ref_. The corresponding cluster areas decrease with *γ*. We name these clusters set 2. At last, another nine clusters emerging from the same growth sequence (realisation) as *C*_ref_ are selected. Together with *C*_ref_, they make up set 3. In addition, ten clusters from different *γ* values are selected according to the criterion that they are close to *C*_ref_ in terms of gross building volume and size. They constitute set 4. See Supplementary Fig. 1 for the cross plot of size versus gross building volume of all selected clusters and some of them depicted.

### 3D UCP data

In order to make use of the gravitational urban growth model output, the grid value *v*_*i*_ in pixel *i* was taken as the floor count of the building in this pixel. The system was aggregated into to a coarser domain of 200 × 200, thus each coarse pixel consists of 25 finer pixels with values {*v*_1_, *v*_2_, . . . , *v*_25_}. Then the urban fraction *f*_u_ of this coarse pixel was calculated as $\frac{N({v}_{i}{\,}> {\,}0)}{25}$, where *N*(⋅) is a function that counts the number of considered values that match the criterion of it. Only coarse pixels with urban fraction no <20% were taken as urban cells^68^, in the end we got a 200 × 200 urban/non-urban matrix for each output. For some outputs from large *γ* values, the *v* values of pixels near the centre become very large after many interations. In order to have more realistic city centres, we applied a threshold of 30 for maximum average number of building storeys on each coarse pixel and rescaled the building height distribution as follow. If $\bar{v}=\frac{\sum {v}_{i}}{N({v}_{i}{\,}> {\,}0)}> 30$, ${v}_{i}=\frac{30{v}_{i}}{\bar{v}}$, thus the average building height $\bar{v}$ for all coarse pixels will not exceed 30 storeys. Then for each coarse pixel marked as urban cell, we calculated the building height distribution {*f*_h1_, *f*_h2_, . . . , *f*_h*j*_} following ${f}_{{\rm{h}}i}=\frac{N({v}_{i}{\,}={\,}i)}{N({v}_{i}{\,} > {\,} 0)}$, where *f*_h*i*_ denotes the share of buildings with height of *i* storeys, and *j* is the maximum value of the original 1000 × 1000 lattice.

We applied the CCA algorithm^69^ on the aggregated urban/non-urban matrix to assign all urban pixels into clusters. In this study, we only focus on the central, largest cluster of each output. In addition, only the central clusters with >200 pixels, which do not touch any edge of the coarse domain were considered.

For each coarser grid cell, the proportion of urban surface occupied by building footprints was calculated^28^ as ${f}_{{\rm{b}}}=\frac{{W}_{{\rm{b}}}}{Y{\,}+{\,}{W}_{{\rm{b}}}}$, where *W*_b_ and *Y* are building width and street width, respectively. The building volume *w* for each grid was calculated according to

4 $$w={A}_{{\rm{grd}}}\times {f}_{{\rm{u}}}\times {f}_{{\rm{b}}}\times \bar{v}\times {H}_{{\rm{f}}},$$

where *A*_grd_ is the area of the grid cell (1 × 1 km^2^ throughout all the simulations), *f*_u_ is urban fraction measured as urban surface fraction, $\bar{v}$ is the aforementioned average building height measured by the number of storeys, and *H*_f_ is the floor height (we assume constant floor height of 3 m for all buildings to further simplify conditions in this study), see the notation in Supplementary Table 3. We take the number of pixels of the considered cluster as the urban size *A*, and the sum of the building volume covered by the cluster as the gross building volume, namely $S = {\sum }{w}_{i}$.

For other parameters required by the DCEP scheme, such as street direction distribution, street width, and building width^28^, we first assumed they are distributed homogeneously within the urban area in order not to introduce further variability. The fraction for each direction (−45^∘^, 0^∘^, 45^∘^, and 90^∘^ to the north clockwise) in each pixel is 25%. For street width *Y* and building width *W*_b_, 20 m and 15 m are taken, respectively. Based on the selected clusters, 50 UCP datasets are created.

We create additional 42 UCP datasets that feature ten spatial patterns (see Supplementary Fig. 3 and Table 1 for examples of each pattern), which are named set 5. For these clusters, we chose a street canyon width of 15 m and building width of 20 m. We take a different street canyon width compared to sets 1–4, since in set 5 some of the clusters are rather small and as smaller canyon width leads to stronger UHI intensities, we have a better signal to noise ratio.

To study how the street canyon width influences the parameters *a*_1_, *a*_2_, *a*_3_ in Eq. (1), we pick five UCP datasets from set 1 and another five from set 3 to create additional UCP data. Based on each of these UCP datasets, we then create four new UCP datasets by only changing the street canyon width to 10 m, 15 m, 25 m and 30 m, respectively, and changing the building width accordingly to keep the fraction of building plan area unchanged. Then for each street width in {10, 15, 20, 25, 30} m, we get ten simulations and fit the results with Eq. (1). Comparing resulting *a*_1_, *a*_2_, *a*_3_, we are able to study the influence of the street width. These modified UCP datasets are together named set 6.

The parameters taken for different UCP datasets can be found in Supplementary Table 4.

### UCP data from different urbanisation scenarios of Berlin

To illustrate how our findings can be used in for real-world cities, we create a series of UCP data based on different hypothetical urbanisation scenarios for Berlin. This is done by modifying the real UCP data taken for the reference run. Assuming that the change of living space only happens in vertical direction, we increased (or decreased) the average building height proportionally by −50%, −25%, +25%, +50%, and +100%. Thus, we get five UCP scenarios with a gross building volume increment of −50%, −25%, 25%, 50%, and 100% relative to the real UCP data.

Similarly, we can also allocate the change by altering the urban fraction. For the scenarios of decreasing gross building volume by 50% and 25%, we simply decrease the urban fraction of each grid cell by 50% and 25%. However, for increasing gross building volume scenarios, we decrease the vegetation surface of each grid cell by the same percentages to get three scenarios with gross building volume increased by 25%, 50%, and 100%. Similar method has been used by Schubert and Grossman-Clarke^28^.

It has to be noted that both vertical changes and horizontal changes are constrained within already urbanised grid cells, that is, without changing the shape of the urban cluster. In the third scenario the change of gross building volume is achieved by modifying the extent of the urban cluster. This is implemented by randomly adding or removing urban grid cells. For decreased gross building volume scenarios, we simply repeated the process of randomly removing an urban grid cell from the edge of the cluster. For increased gross building scenarios, the following steps are repeated until the gross building volume of the expanded urban cluster approximately agrees with the desired size. Step 1: randomly pick a grid cell from the urban cluster; step 2: randomly select a non-urban grid cell that is adjacent to an urban grid cell; and step 3: replace this non-urban cell with the urban cell selected by step 1. At the end, we get ten scenario UCP datasets with gross building volume changed by −50%, −25%, −10%, +10%, +25%, +40%, +50%, +60%, +75%, and 100% respectively,

### Reporting summary

Further information on research design is available in the Nature Research Reporting Summary linked to this article.

## Supplementary information

Supplementary Information

Reporting Summary

## Data Availability

The initial lateral and boundary conditions for the simulations were obtained from the ERA-Interim data (https://www.ecmwf.int/en/forecasts/datasets/reanalysis-datasets/era-interim). The UCP data and hourly 2-m temperature data of all simulations can be accessed at “Urban canopy parameterisation data for urban climate simulation using CCLM/DCEP and hourly 2-m temperature output (10.1594/PANGAEA.914906)”^70^. Further data are available from the corresponding author upon reasonable request.

## Code availability

The source code of the COSMO-CLM model is available from the CLM-Community (clm-community.eu) after registration. The DCEP code is available from S. Schubert upon request. The R-package dcepucp used to create UCP datasets for the simulations is available at https://github.com/sebschub/dcepucp. Custom R-code may be provided upon request.
