# Supplementary material for: On the influence of density and morphology on the Urban Heat Island intensity
Source: Nat Commun. 2020 May 27;11:2647. doi: 10.1038/s41467-020-16461-9 (PMC7253412; doi:10.1038/s41467-020-16461-9)
Supplement: Supplementary file 1 — Supplementary Information [file 41467_2020_16461_MOESM1_ESM.pdf]

On the influence of density and morphology on the Urban Heat Island intensity

Supplementary Information

Yunfei Li[1,2], Sebastian Schubert[1,3], Jürgen P. Kropp[1,2], & Diego Rybski[1,4*]

[1]*Potsdam Institute for Climate Impact Research – PIK, Member of Leibniz Association, P.O. Box 60 12 03, Potsdam, 14412, Germany.*

[2]*Institute for Environmental Science and Geography, University of Potsdam, Am Neuen Palais 10 14469 Potsdam, Germany.*

[3]*Geography Department, Humboldt-Universität zu Berlin, Unter den Linden 6, 10099 Berlin, Germany.*

[4]*Department of Environmental Science Policy and Management, University of California Berkeley, 130 Mulford Hall #3114, Berkeley, CA 94720, USA.*

[*]*To whom corresponce should be addressed: ca-dr@rybski.de*

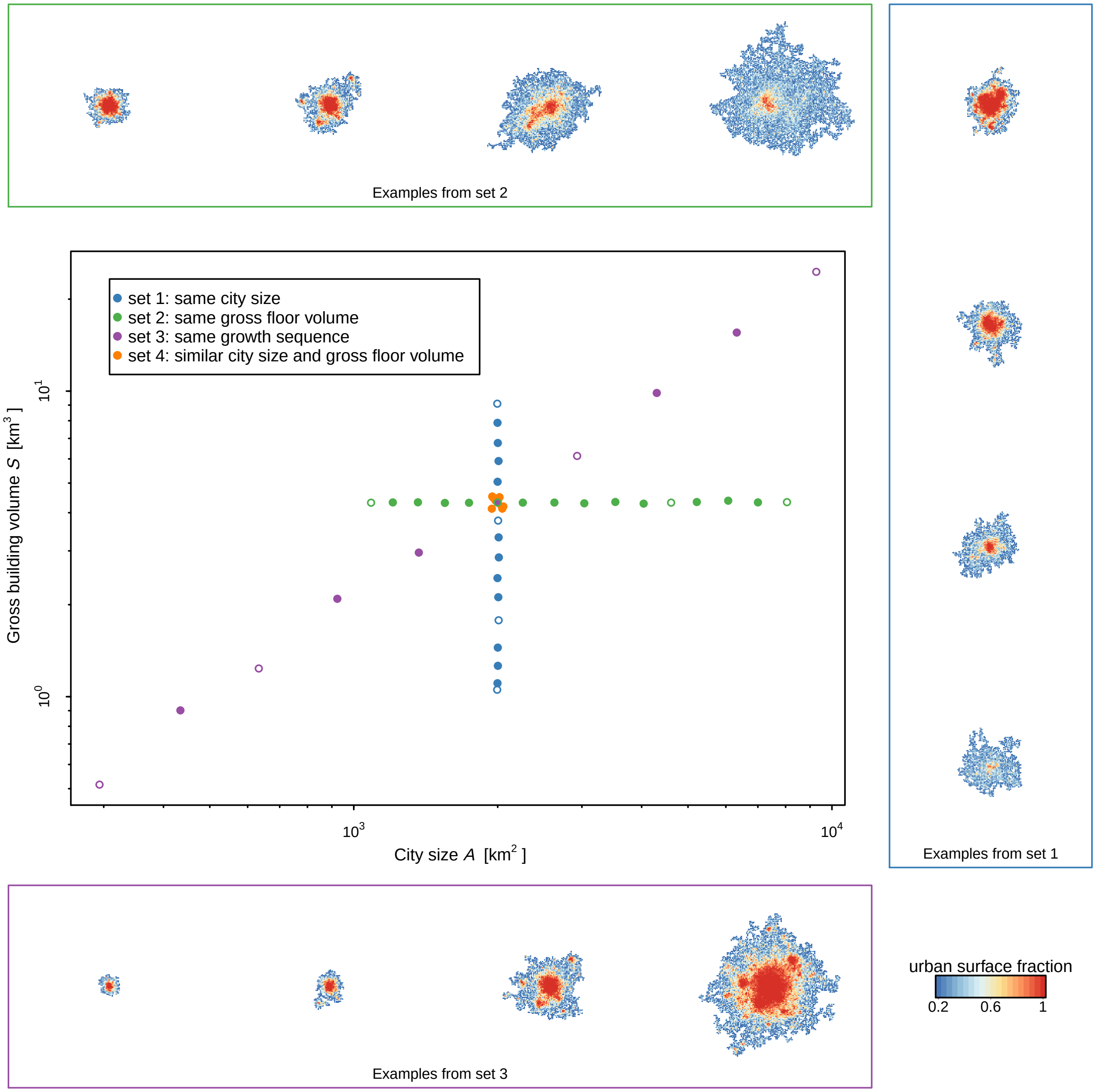

Supplementary Figure 1: Examples of analysed urban clusters. From the large number of previously generated urban clusters[1], 50 haven been selected which are illustrated here. The clusters are organised into sets according to the criteria they have been chosen by, i.e. same area (set 1, blue), same gross building volume (set 2, green), same growth sequence (set 3, violet), and similar size and similar gross building volume (set 4, orange). The surrounding panels display 4 examples each and the colour scale indicates the urban fraction in each urban site.

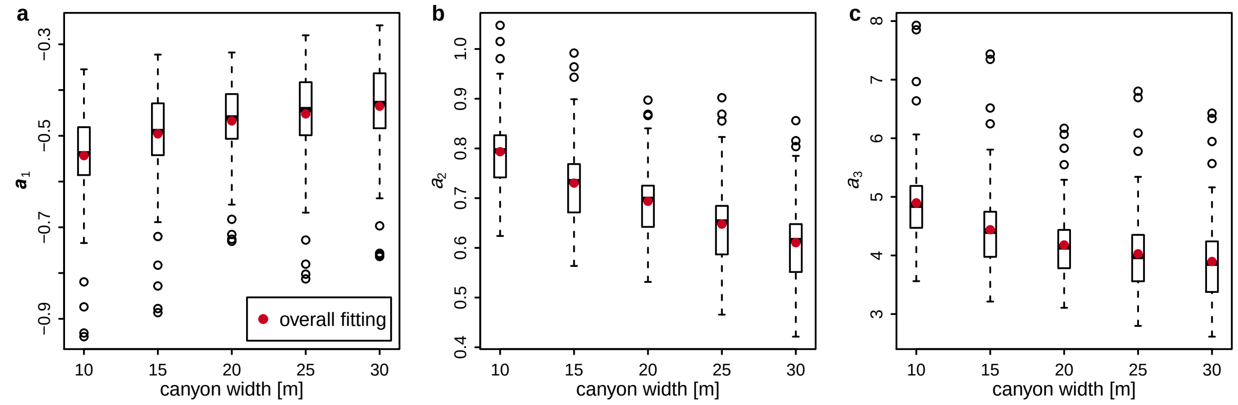

Supplementary Figure 2: Parameters from Eq. (1) depending on the canyon width. In order to assess the influence of the canyon width on the pre-factors in Eq. (1) we run the urban climate simulations for 10 generated urban clusters (see Supplementary Fig. 1) and repeat for different canyon widths between 10 m and 30 m. The panels show box plots of the parameters vs. the canyon width, i.e. for a_1 in **a**, for a_2 in **b**, and for a_3 in **c**. The spreading is obtained from bootstrapping (200 repetitions) and the red dots represent the estimated parameters without bootstrapping. In the boxes, centre line indicates median; box limits are upper and lower quartiles; whiskers denote 1.5x interquartile range; points are outliers.

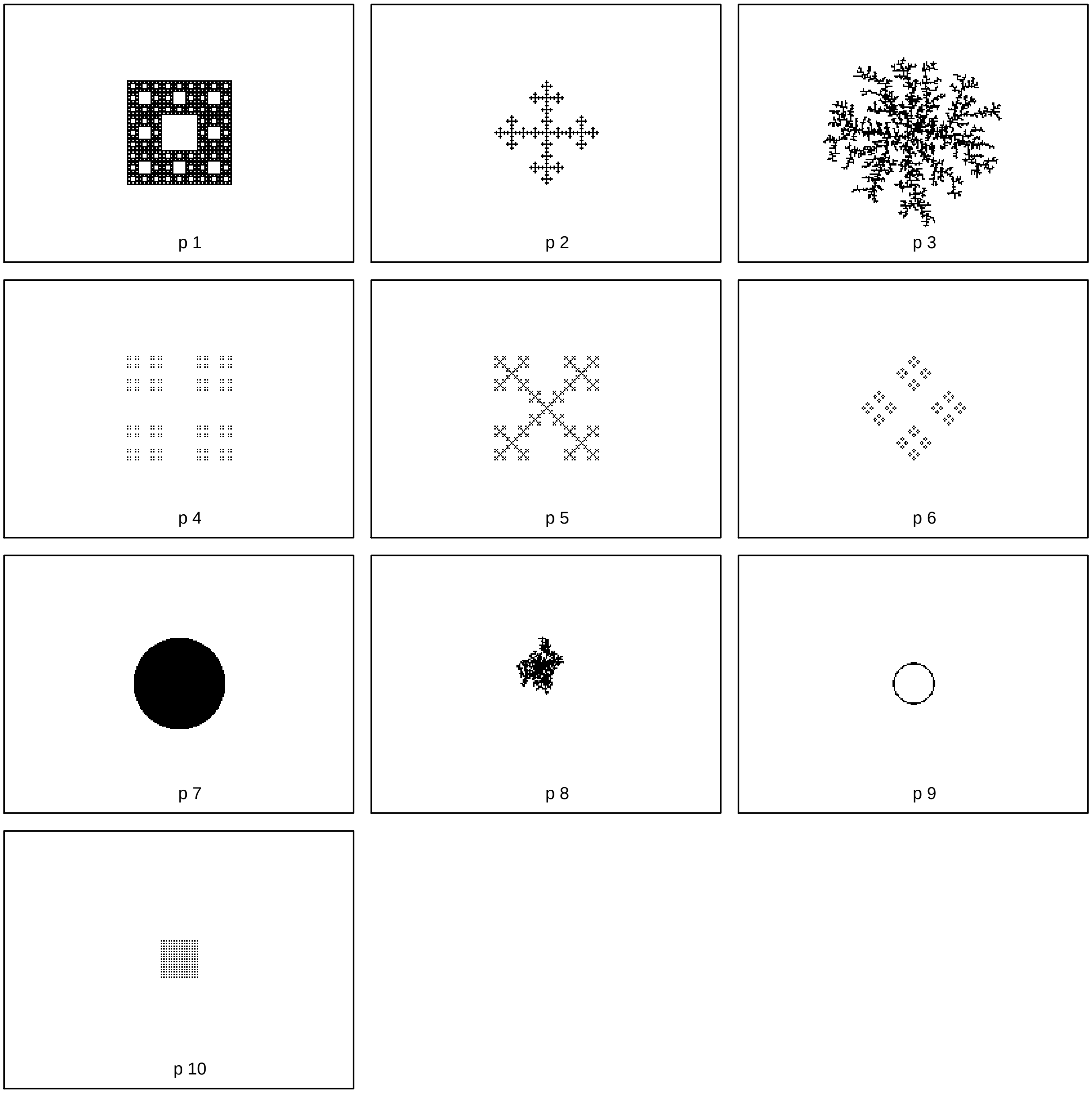

Supplementary Figure 3: Examples of analysed spatial patterns in set 5. Different sizes of these patterns were used for the urban climate simulations. Details on the patterns are provided in Supplementary Table 1. The building volume in each pixel is set constant throughout the patterns. Pattern 2 resembles the Ville Contemporaine as envisioned by Le Corbusier; pattern 9 resembles the Apple Park in Cupertino, California.

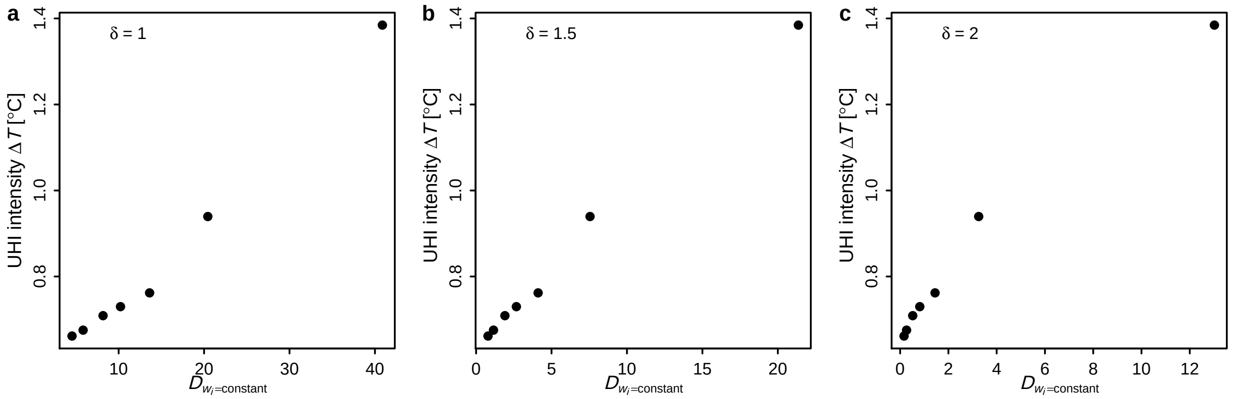

Supplementary Figure 4: Estimation of exponent δ in Eq. (2). For pattern 10 (see Supplementary Fig. 3) with variable spacing and constant size we plot the UHI intensity ΔT as a function of $D_{w_i=\text{const.}} = \frac{1}{N}\sum\sum_{i,j}^{N,N} d_{i,j}^{-\delta}$ for $\delta = 1$ (panel **a**), $\delta = 1.5$ (panel **b**), and $\delta = 2$ (panel **c**). For $\delta = 1.5$ we find an approximately linear relation implying that D linearly relates to the UHI intensity given constant number of urban sites and only capturing the spatial organization.

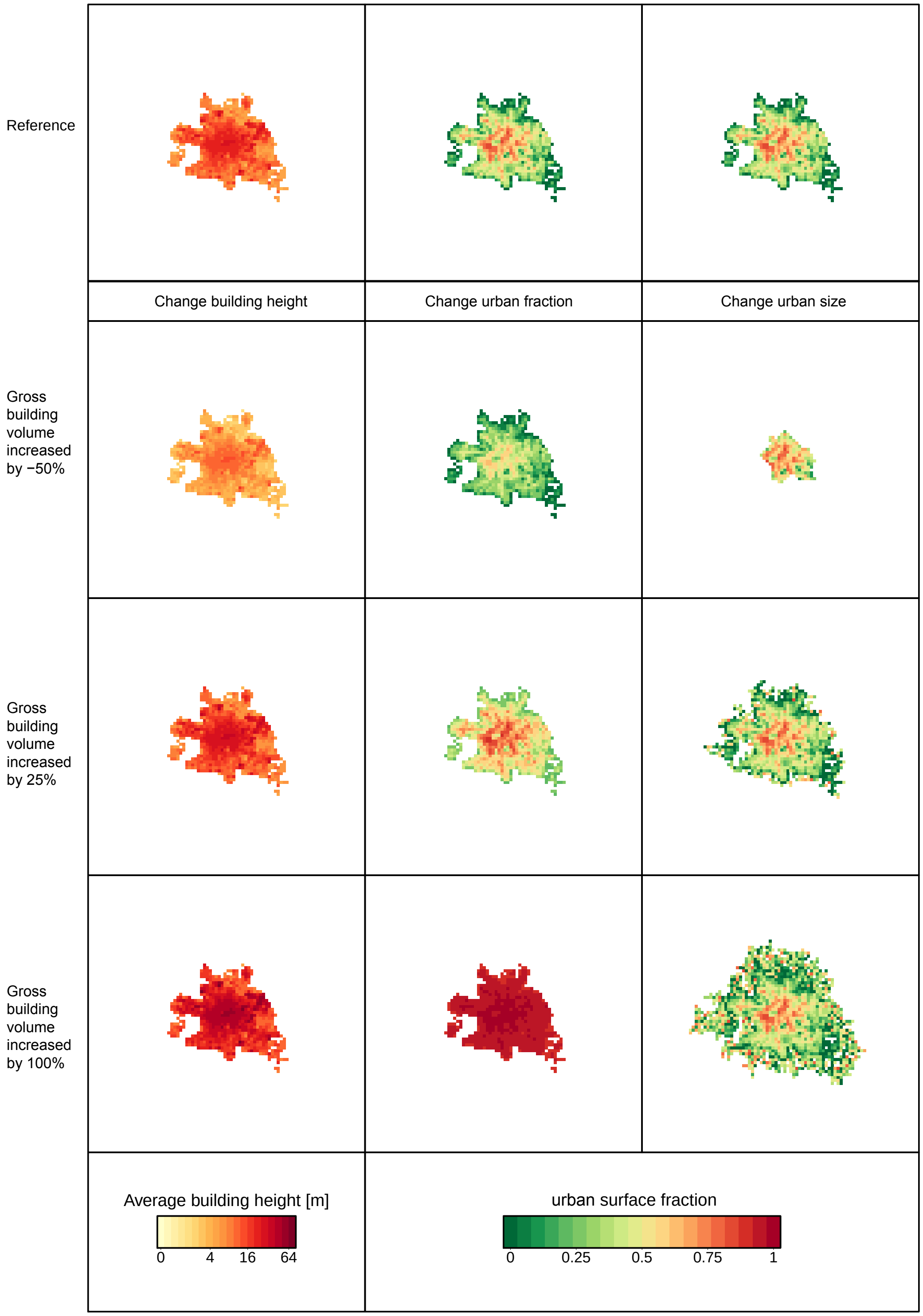

Supplementary Figure 5: Visualisation of some UCP datasets from different urbanisation scenarios based on the UCP dataset of Berlin. The first column shows the average building height map, the second and third show the urban surface fraction maps, respectively. Panels in the first row are from real UCP dataset of Berlin used in the reference run, whereas Panels in other rows are from UCP datasets where gross building volume is increased by -50%, +25%, and +100%.

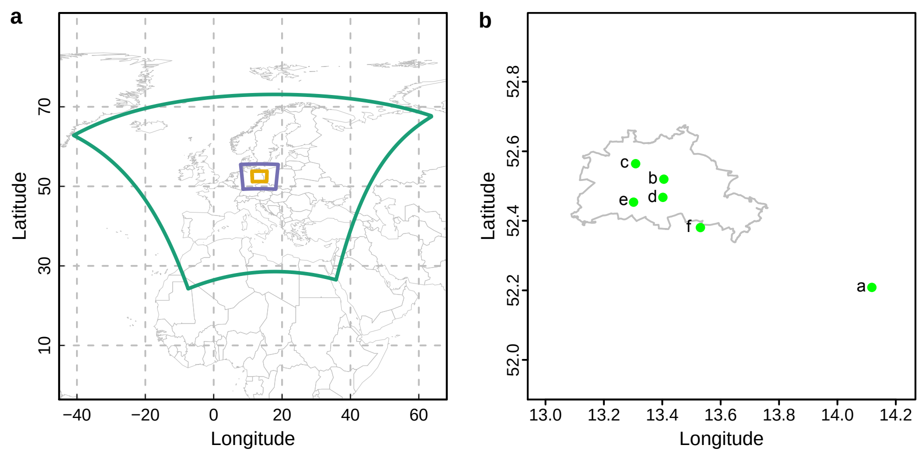

Supplementary Figure 6: Model domains and location of weather stations. a, Nested domains with resolution of 0.165° (aqua green), 0.025° (purple), 0.009° (orange). **b,** Green points indicate locations of weather stations, a) Lindenberg, b) Alexanderplatz, c) Tegel, d) Tempelhof, e) Dahlem, f) Schönefeld, grey line is the administrative boundary of Berlin city.

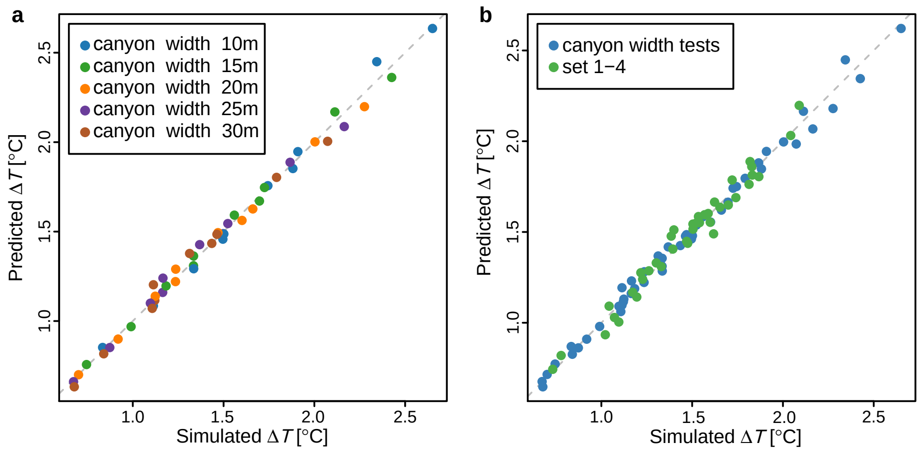

Supplementary Figure 7: Fitting performances of Eq. (5). a, Fitting for varying canyon width tests (see Methods and Supplementary Information). **b**, Fitting for both canyon width tests and simulations with clusters in Supplementary Fig. 1.

Supplementary Table 1: Information about the spatial patterns for which the UHI intensities have been simulated and fitted by Eq. (2).

pattern	number	size A (km^2)	fractal dimension	extra information
p 1	3	64, 512, 4096	1.89	Sierpinski Carpet
p 2	3	25, 125, 625	1.46	Vicsek fractal
p 3	5	102, 256, 625, 1026, 4096	≈1.71[2]	DLA[2–6] cluster
p 4	3	16, 64, 256	1.26	Cantor Dust
p 5	3	25, 125, 625	1.46	diagonal Vicsek fractal
p 6	3	16, 64, 256	1.26	diagonal Cantor Dust
p 7	8	25, 129, 621, 997, 1505, 2001, 3001, 4001	2 (small scales)	circle
p 8	2	624, 626	≈1.46*, 1.72*	gravitational model[1]
p 9	5	28, 124, 624, 1004, 2004	1 (small scales)	ring
p 10	7	225, 225, 255, 225, 225, 225, 225	-	space between neighbour cells: 0, 1, 2, 3, 4, 6, 8

*) We list the abbreviation, the number of realisations, the size, the fractal dimension (* indicate measured values), and additional information. The patterns are illustrated in Supplementary Fig. 3.

Supplementary Table 2: 2m air temperature [K] statistics of the reference run against observational data.

	Lind.	Alex.	Tegel	Temp.	Dahlem	Schön.
ME	-0.19	-0.51	-0.77	0.42	0.77	0.58
MAE	0.77	0.74	1.04	1.02	1.45	0.81
RMSE	0.94	0.93	1.26	1.27	1.85	1.07

Supplementary Table 3: Notation table.

notation	meaning	unit
S	city gross building volume	km^3
A	urban area	km^2
ΔT	urban heat island intensity	K
w_i	building volume of grid i	km^3
d_{ij}	distance between grid i, j	km
N	number of urban grid cells	-
v_i	value of cell i in gravitational model output	-
f_u	urban surface fraction	-
f_b	share of building plan area in urban surface	-
W_b	building width	km *
Y	street canyon width	km *
$\bar{v}$	average building height	storey
A_grd	area of simulation grid cell	km^2
H_f	height per floor, 0.003km *	-
γ	parameter in the gravitational model	-
B_i	building plan area in grid cell i	km^2
H_i	average building height in grid cell i	km *
λ_i	urban street canyon aspect ratio in grid cell i	-

*) We use km instead of m because otherwise the parameters of Eq. (3) would become very small.

Supplementary Table 4: Information about UCP parameters taken in different simulations.

Exp. series	gravitational clusters	spatial patterns	canyon width tests
UCP data-set	set 1, 2, 3, 4	set 5	set 6
number of simulation	50	42	50
description	Suppl. Fig. 1	Suppl. Fig. 3	5 from set 1,<br>5 from set 3
urban fraction f_{u}	0 - 1	0.53	0 - 1
average building height [m]	3 - 90	8.63	3 - 90
street canyon Y [m]	20	15	10,15,20,25,30
building width W_{b} [m]	15	20	$W_{\mathrm{b}}/Y = 15/20$
regression applied	Eq. (1), (3), (5)	Eq. (2)	Eq. (3), (5)

Supplementary Notes 1: Introduction of street canyon width and aspect ratio.

If we include B_i, H_i, λ_i as building footprint area, average building height, and street canyon aspect ratio, respectively, for grid cell i (Supplementary Table 3), then they are related by $w_i = B_i H_i$, $\lambda_i = H_i / Y_i$, and we can further rewrite D in Eq. (3) as:

$$D = \frac{1}{N} \sum_{j}^{N} \sum_{i \neq j}^{N} (f_{\mathrm{u}i} B_i \lambda_i)^{1/2} d_{ij}^{-3/2} \, , \tag{5}$$

Fitting Eq. (5) leads to identical to those of Eq. (3).

Supplementary References

[1] Li, Y., Rybski, D. & Kropp, J. P. Singularity cities. *Environ. Plan. B* **Online First**, https://doi.org/10.1177/2399808319843534 (2019).

[2] Batty, M. & Longley, P. *Fractal Cities: A Geometry of Form and Function* (Academic Press Inc, San Diego, CA and London, 1994).

[3] Witten Jr., T. A. & Sander, L. M. Diffusion-limited aggregation, a kinetic critical phenomenon. *Phys. Rev. Lett.* **47**, 1400–1403 (1981).

[4] Fotheringham, A. S., Batty, M. & Longley, P. A. Diffusion-limited aggregation and the fractal nature of urban growth. *Pap. Reg. Sci. Assoc.* **67**, 55–69 (1989).

[5] Batty, M., Longley, P. & Fotheringham, S. Urban growth and form: scaling, fractal geometry, and diffusion-limited aggregation. *Environ. Plan. A* **21**, 1447–1472 (1989).

[6] Batty, M. *The New Science of Cities* (MIT Press, Cambridge, MA, 2013).
