# Supplementary material for: On the influence of density and morphology on the Urban Heat Island intensity
Source: Nat Commun. 2020 May 27;11:2647. doi: 10.1038/s41467-020-16461-9 (PMC7253412; doi:10.1038/s41467-020-16461-9)
Supplement: Supplementary file 2 — Reporting Summary [file 41467_2020_16461_MOESM2_ESM.pdf]

nature research

Corresponding author(s): Dr. Diego Rybski
Last updated by author(s): Apr 7, 2020

Reporting Summary

Nature Research wishes to improve the reproducibility of the work that we publish. This form provides structure for consistency and transparency in reporting. For further information on Nature Research policies, see Authors & Referees and the Editorial Policy Checklist.

Statistics

For all statistical analyses, confirm that the following items are present in the figure legend, table legend, main text, or Methods section.

n/a	Confirmed	
☐	☒	The exact sample size (n) for each experimental group/condition, given as a discrete number and unit of measurement
☐	☒	A statement on whether measurements were taken from distinct samples or whether the same sample was measured repeatedly
☐	☒	The statistical test(s) used AND whether they are one- or two-sided<br>*Only common tests should be described solely by name; describe more complex techniques in the Methods section.*
☐	☒	A description of all covariates tested
☒	☐	A description of any assumptions or corrections, such as tests of normality and adjustment for multiple comparisons
☐	☒	A full description of the statistical parameters including central tendency (e.g. means) or other basic estimates (e.g. regression coefficient) AND variation (e.g. standard deviation) or associated estimates of uncertainty (e.g. confidence intervals)
☒	☐	For null hypothesis testing, the test statistic (e.g. F, t, r) with confidence intervals, effect sizes, degrees of freedom and P value noted<br>*Give P values as exact values whenever suitable.*
☒	☐	For Bayesian analysis, information on the choice of priors and Markov chain Monte Carlo settings
☒	☐	For hierarchical and complex designs, identification of the appropriate level for tests and full reporting of outcomes
☒	☐	Estimates of effect sizes (e.g. Cohen's d, Pearson's r), indicating how they were calculated

Our web collection on statistics for biologists contains articles on many of the points above.

Software and code

Policy information about availability of computer code

Data collection	cosmo_5.0_clm9 model and online tool WebPEP for external data preparation are available from CLM-Community(clm-community.eu) after registration; dcepucp R package used to create UCP data set for the simulations is avaliable at https://github.com/sebschub/dcepucp; custom R code running on R3.34 may be provided upon request.
Data analysis	custom R code (running on R3.34) for post-processing and data analysis may be provided upon request.

For manuscripts utilizing custom algorithms or software that are central to the research but not yet described in published literature, software must be made available to editors/reviewers. We strongly encourage code deposition in a community repository (e.g. GitHub). See the Nature Research guidelines for submitting code & software for further information.

Data

Policy information about availability of data

All manuscripts must include a data availability statement. This statement should provide the following information, where applicable:

- Accession codes, unique identifiers, or web links for publicly available datasets
- A list of figures that have associated raw data
- A description of any restrictions on data availability

Data availability
All Urban Canopy Parameter (UCP) data and hourly 2 meter temperature data of all simulations can be accessed freely at 'Urban canopy parameterization data for urban climate simulation usingCCLM/DCEP and hourly 2m temperature output [https://doi.org/10.1594/PANGAEA.914906]'.

Field-specific reporting

Please select the one below that is the best fit for your research. If you are not sure, read the appropriate sections before making your selection.

☐ Life sciences ☐ Behavioural & social sciences ☒ Ecological, evolutionary & environmental sciences

For a reference copy of the document with all sections, see nature.com/documents/nr-reporting-summary-flat.pdf

Ecological, evolutionary & environmental sciences study design

All studies must disclose on these points even when the disclosure is negative.

Study description	We run high resolution CCLM/DCEP climate simulation with different urban structures under the same weather condition to quantify the effect of urban size, urban building volume and urban form on the urban heat island effect.
Research sample	We used on-line tool WebPEP (clm-community.eu) to create the external data for the simulations;<br>The initial lateral and boundary condition for the simulations are from the ERA-Interim data (https://www.ecmwf.int/en/forecasts/datasets/reanalysis-datasets/era-interim). The observational data from weather stations are available at https://opendata.dwd.de/climate_environment/CDC/observations_germany/.
Sampling strategy	n.a.
Data collection	n.a.
Timing and spatial scale	n.a.
Data exclusions	n.a.
Reproducibility	Open-source datasets and model, tools were used in this study. Input urban canopy parameterization (UCP) datasets, and parameterizations were specified in the methods part. The simulation output data and UCP datasets as well as the detailed methods details are provided.
Randomization	n.a.
Blinding	n.a.

Did the study involve field work? ☐ Yes ☒ No

Reporting for specific materials, systems and methods

We require information from authors about some types of materials, experimental systems and methods used in many studies. Here, indicate whether each material, system or method listed is relevant to your study. If you are not sure if a list item applies to your research, read the appropriate section before selecting a response.

Materials & experimental systems

n/a	Involved in the study
☒	☐ Antibodies
☒	☐ Eukaryotic cell lines
☒	☐ Palaeontology
☒	☐ Animals and other organisms
☒	☐ Human research participants
☒	☐ Clinical data

Methods

n/a	Involved in the study
☒	☐ ChIP-seq
☒	☐ Flow cytometry
☒	☐ MRI-based neuroimaging
